# Optimization of Commercial Microwave Assisted-Extraction Conditions for Recovery of Phenolics from Lemon-Scented Tee Tree (*Leptospermum petersonii*) and Comparison with Other Extraction Techniques

**DOI:** 10.3390/foods11010050

**Published:** 2021-12-26

**Authors:** Md Saifullah, Taiwo Olusesan Akanbi, Rebecca McCullum, Quan Van Vuong

**Affiliations:** 1 School of Environmental and Life Sciences, College of Engineering, Science and Environment, The University of Newcastle, Ourimbah, NSW 2258, Australia; md.saifullah@uon.edu.au (M.S.); taiwo.akanbi@newcastle.edu.au (T.O.A.); rebecca.richmond@uon.edu.au (R.M.); 2 Department of Agro Product Processing Technology, Faculty of Applied Science and Technology, Jashore University of Science and Technology, Jashore 7408, Bangladesh

**Keywords:** polyphenol, antioxidant, lemon-scented tea tree, extraction, method comparison, MAE, UAE

## Abstract

The lemon-scented tea tree (LSTT) is an Australian native herb and is a rich source of essential oil and phenolics. The ETHOS X extraction system is known as a commercial microwave-assisted extraction (MAE) system for extracting bioactive compounds from plant materials. This study investigated the influence of soaking time, radiation time, microwave power, and sample to solvent ratio on the extraction efficiency of polyphenols and antioxidant properties from lemon-scented tea tree leaves and optimized the extraction conditions using response surface methodology (RSM). The effectiveness of ETHOS X was further compared with ultrasound-assisted extraction (UAE) and shaking water bath (SWB) techniques. The results revealed that soaking time did not significantly affect the recovery of phenolics from the leaves (*p* > 0.05). Thus, soaking is not required for the ETHOS X extraction of polyphenols from LSTT leaves. RSM was successfully applied to explore the impact of ETHOS X extraction conditions and optimize the extraction conditions. Radiation time significantly affects the recovery yield of phenolics (*p* < 0.05) positively, whereas irradiation power and sample to solvent ratio adversely influenced the extraction yields of phenolics. The optimal ETHOS X extraction conditions were: radiation time of 60 min, irradiation power of 600 W, and sample to solvent ratio of 2 g/100 mL. Under these conditions, 119.21 ± 7.09 mg of phenolic, 85.31 ± 4.55 mg of flavonoids, and 137.51 ± 12.52 mg of proanthocyanidins can be extracted from a gram of dried LSTT leaves. In comparison with UAE and SWB, ETHOS X is not more effective for the extraction of phenolics than UAE and SWB. However, this technique can save half of the solvent volume compared to UAE and SWB techniques.

## 1. Introduction

Phytochemicals derived from plant materials have been used as the key ingredient in many foods, nutraceuticals, and pharmaceuticals products [1]. Of those, polyphenols are known as a major group that has been linked with numerous health benefits. Polyphenols have been widely applied in the food, pharmaceutical, and cosmetic industries and have anti-inflammatory, antimicrobial, and anti-aging properties, among other health benefits [2,3]. Therefore, it is important to effectively extract and isolate phenolics from plant materials using optimal conditions and suitable techniques [4,5]. Several traditional, novel extraction, and a combination of modern and conventional extraction techniques have been applied to extract phenolics from plant materials [6,7]. However, the suitability and selection of the right extraction method and conditions vary depending on the type/nature of the sample, yield of targeted chemical compounds, and cost of extraction. 

**Figure 1:**
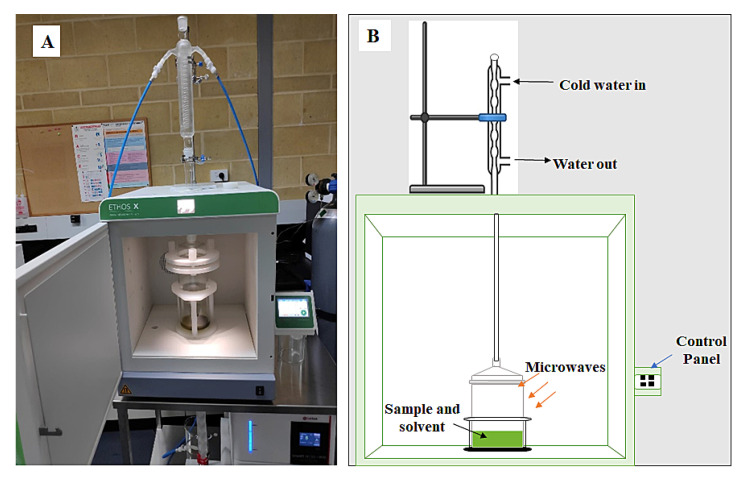
ETHOS X advanced microwave-assisted extraction system (**A**), experimental process, and scheme of the MAE system applied in this study (**B**).

MAE is one of the most common advanced techniques applied for the extraction of plant phytochemicals [8,9]. In this technique, microwaves generate heat and create a pressure gradient in the sample and plant phytochemicals are released through diffusion, bursting, or rupturing the tissue and cell wall [10]. The rising temperature also helps to soften the plant tissue, increase mass transfer, heat transfer, and solvent penetration in the sample, break down the structure of chemicals, and help to release polyphenols in the solvent. There are several factors involved in MAE, which may influence the quantity and quality of phytochemicals extracted from plant materials. However, solvent ratio, microwave power level, and irradiation time are the most common parameters [7,8,11]. The range of various extraction parameters in MAE can be varied according to the sample, which are mainly selected based on preliminary experiments. Esquivel-Hernández et al. [12] and Kala et al. [13] reported a wide range of extraction parameters values, i.e., microwave power (17.8 W–1000 W) and extraction time (10 s–5 h). The ETHOS X extraction system has been widely used recently for extracting phytochemicals from plant parts (Figure 1). It is recognized as a commercial microwave system, which has a closed vessel attached with a reflux unit to control the pressure by condensing the vaporized solvent [14]. The extraction efficiency of bioactive compounds in ETHOS X extraction system is affected by various extraction parameters. Therefore, optimizing the ETHOS X conditions for the extraction of plant phytochemicals is essential. Among the optimization techniques, response surface methodology (RSM) is a very effective tool for the prediction and optimization of the extraction conditions [15,16]. 

The LSTT is well known for its strong lemon flavor. It has been used as traditional medicine and in tea blends and is also applied as a natural preservative in food products and as a substitute for lemon flavor in dairy products [16,17,18]. The leaves have been mainly used for the extraction of essential oil. However, the leaves also contain a good quantity of polyphenols with strong antioxidant, antimicrobial activities, which could be of great interest and can be used for food and nutraceuticals products. It should be noted that a large quantity of leaves has been discarded or used for mulching after the distillation of essential oils. These leaves also contain high levels of phenolic compounds, which can be recovered for further applications. We previously optimized ultrasound-assisted extraction (UAE) conditions and investigated the extraction yield of conventional extraction techniques (shaking water bath (SWB)) in liquid crude extracts [19]. However, the dry extract is more stable, easy to handle, and extends the application possibilities in dry form. Therefore, it would be worth optimizing the commercial MAE conditions for phenolics and antioxidants from lemon-scented tea tree leaves and comparing its effectiveness with UAE and SWB in terms of recovery yields in the dry form of extract. This study investigated the impact of MAE parameters, including soaking time, microwave power, and radiation time, and sample to solvent ratio on the phenolics and antioxidant level in LSTT extract then optimized the commercial MAE conditions for recovery of phenolics and finally compared its effectiveness with UAE and SWB. These conditions can be applied for the recovery phenolic compounds from LSTT leaves, known as waste, generated from essential oil production. 

## 2. Methodology

### 2.1. LSTT Leaves Harvesting and Preparation for Extraction

The leaves were arbitrarily collected from the trees on the central coast, Ourimbah, NSW, Australia (latitude of 33.4° S, longitude of 151.4° E) in February 2020. After collection, the leaves were immediately transferred to the laboratory, and soaked into liquid nitrogen. The frozen leaves were freeze dried for 48 h using a freeze dryer (Bench Top Pro BTP-3ESE0X, Philadelphia, PA, USA). The moisture content of the dried ground leaves was 3.2%. A commercial blender (John Morris Scientific, Chatswood, NSW, Australia) was used to grind the dried leaves. The ground leaves were sieved through a steel standard sieve mesh of 1.4 mm. The fine samples collected after sieving were kept in a labelled, airtight packet at −20 °C for extraction.

### 2.2. Chemical and Reagent for Different Assays

All of the solvents, chemicals, and reagents used in this study were analytical grade. The organic solvents (acetone, ethanol, and methanol), vanillin, and sodium hydroxide were obtained from Merck (Darmstadt, Germany). Folin-Ciocalteu’s reagent and other chemicals including anhydrous sodium carbonate, sodium nitrite, hydrochloric acid, potassium persulfate, copper (II) chloride, ferric chloride, aluminum chloride hexahydrate, ammonium acetate, 2,2-diphenyl-1-picrylhydrazyl (DPPH), 2,2-Azino-bis (3-ethylbenzothiazoline-6-sulfonic acid) diammonium salt (ABTS), (±)-6-hydroxy-2,5,7,8-tetramethylchroman-2- carboxylic acid(Trolox), 2,4,6-Tri (2-pyridyl)-s-triazine, neocuproine, gallic acid, and catechin were purchased from Sigma-Aldrich Pty Ltd. (Castle Hill, Sydney, Australia)

### 2.3. Experimental Design

**Figure 2:**
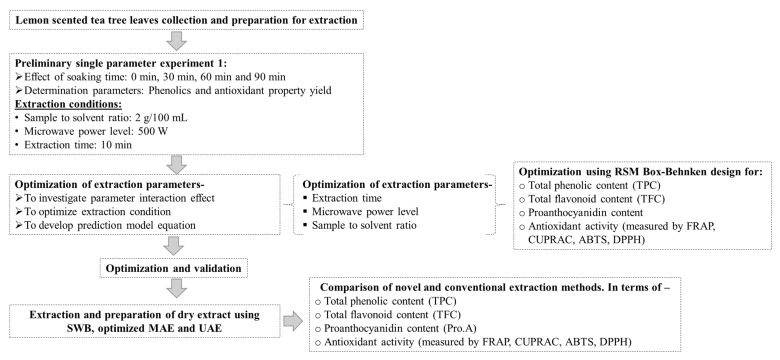
Experimental flow diagram for the study. Here, Ferric reducing antioxidant power (FRAP), Cupric ion reducing antioxidant capacity (CUPRAC), ABTS radical scavenging assay (ABTS), DPPH free radical scavenging assay (DPPH).

The experimental design for this study is shown in Figure 2. Soaking is the process whereby the dried sample is wet thoroughly with the solvent before the microwaving process. In this study, various soaking times, ranging from 0 min (control), 30 min, 60 min, and 90 min was investigated. Aqueous acetone (50% *v*/*v*) was used as the extraction solvent since a previous study found that it was the most effective solvent for extracting polyphenols and antioxidant potentials from lemon-scented tea tree leaves [19]. Two grams of dry ground leaves were added into 100 mL of solvent in the extraction chamber and soaked for different soaking times before microwave extraction at 500 W for 10 min. In RSM optimization, radiation time (40 min, 50 min, and 60 min), microwave power level (600 W, 800 W, and 1000 W), and sample to solvent ratio (2 g/100 mL, 4 g/100 mL, and 6 g/100 mL) were selected to investigate the effects of MAE conditions on recovery of phenolics and antioxidant capacity. The independent variables range was selected based on preliminary studies (data not presented in this manuscript). A commercial ETHOS X microwave-assisted extraction system (Milestone, ETHOS X, Sorisole, Italy) was applied for extraction. The experimental design is shown in Table 1. After completion of the extraction, the extract was immediately transferred to 50 mL centrifuge tubes and cooled down for 10 min on ice. The filtrate was centrifuged for 10 min at 2500 rpm followed by filtered through a syringe filter 0.45 µm (Phenomenex Australia Pty. Ltd., Lane Cove West, NSW, Australia) and finally kept at −20 °C for analysis.

The Box–Behnken three-factor, a three-level design involving fifteen experimental runs, was employed (Table 1) including three central points; Box-Behnken is an efficient and economical design [20]. The linear, quadratic, and interaction effects of extraction time (X_1_: 40–60 min), microwave power level (X_2_: 600–1000 W), and sample to solvent ratio (X_3_: 2–6 g/mL) were assessed using RSM. The predicted yield of phenolics and antioxidants in response to extraction parameter level can be represented by this polynomial functional equation (Equation (1)):

(1) $$Y=\beta_{0}+\sum_{i=1}^{n}\beta_{i}X_{i}+\sum_{\begin{array}{c} i=j\\ i<j \end{array}}^{n-1}\sum_{j=2}^{n}\beta_{ij}X_{i}X_{j}+\sum_{i=1}^{n}\beta_{ii}X_{i}^{2}$$

Here, *Y* is the predicted response for phenolics and antioxidant capacities, *β*_0_ is coefficient for intercept, *β_i_*, *β_ii_*, and *β_ij_* represent the regression coefficients of the linear, quadratic, and interaction effects, respectively; *n* is the number of variables; and *X_i_* and *X_j_* are the independent variables [21]. After optimization through RSM modelling and predictions, an experiment was conducted using predicted MAE extraction parameters values. The experimental extraction yield of polyphenols and antioxidant properties were compared with predicted values to assess the precision of the optimization. 

Finally, the optimized MAE conditions were used to prepare a dry phenolic extract for comparison with UAE and SWB. The liquid extracts from MAE, UAE, and SWB were concentrated using a rotavapor (Buchi R-114, Flawil, Switzerland) and followed by freeze dying (Bench Top Pro BTP-3ESE0X, Philadelphia, PA, USA). The dry extracts were diluted using 50% aqueous acetone for the analysis of polyphenols and antioxidant potentials, and the methods were compared based on the level of phenolics and antioxidant potentials of the dry extracts.

### 2.4. Ultrasound-Assisted and Shaking Water Bath Extraction and Preparation

The UAE and SWB extraction were conducted using conditions described by Saifullah et al. [19]. After extraction, the extracts were concentrated using a rotavapor (Buchi R-114, Switzerland) and dried using the freeze drier (Bench Top Pro BTP-3ESE0X, Philadelphia, PA, USA) for comparison with MAE derived lemon scented tea tree dry extract.

### 2.5. Phytochemical Assays

The quantitative assays of different phenolic groups in the LSTT leaves extract were performed using the UV (Ultraviolet–visible) spectrophotometric methods (using a UV spectrophotometer (Cary 60 Bio, UV-Vis, Petaling Jaya, Malaysia)) as described in previously reported protocols. Total phenolic content (TPC) analysis was performed as described by AOCS [22]. The TPC results were expressed as mg gallic acid equivalent per g of dry sample since gallic standard curved was used to measure the TPC. Total flavonoid (TFC) and proanthocyanidin (Pro.A) content were assays according to Zhuang et al. [23] and Sun et al. [24], respectively. For both TFC and Pro.A, the results were expressed as mg catechin equivalent per g dry sample since catechin was used to create a standard curve for TFC and Pro.A. The absorption of light was measured at 765 nm, 510 nm, and 500 nm for TPC, TFC, and Pro.A, respectively. Data acquisition was performed at least thrice for each assay. 

### 2.6. Antioxidant Activity Analysis

Antioxidant properties of the extract were measured by FRAP, CUPRAC, DPPH, and ABTS antioxidant assays. FRAP, DPPH, and ABTS were assayed by following the method described by Benzie and Strain [25], Brand-Williams [26], and Arnao et al. [27], respectively, and CUPRAC was assayed according to the method reported by Apak et al. [28]. The absorbance was measured at the wavelength of 593 nm, 450 nm, 515 nm, and 734 nm for FRAP, CUPRAC, DPPH, and ABTS, respectively, using a UV spectrophotometer (Cary 60 Bio, UV-Vis, Petaling Jaya, Malaysia). Trolox was used to establish the standard curve for antioxidant assays and the results were expressed as mM Trolox equivalent per g sample dry weight of the sample.

### 2.7. Statistical Analysis

To observe the effect of soaking time, all pair mean comparison Tukey-Kramer HSD post hoc test was carried out using JMP software (Version 14.1, SAS Institute Inc., Cary, NC, USA). RSM with Box-bhenken design was applied for the experimental design, analysis of variance, and interaction effect analysis, and prediction of optimal conditions using the JMP software. The JMP software was also used to compare the predicted values with the experimental values using a paired comparison analysis. In all assays, the *p*-value less than 0.05 (*p* < 0.05) was considered statistically significant. All of the experiments/data acquisition were conducted in triplicates and the results are expressed as mean ± standard deviation.

## 3. Results and Discussion

### 3.1. Effect of Soaking Time on the Extraction Yield of Phenolics and Antioxidant Capacities

**Figure 3:**
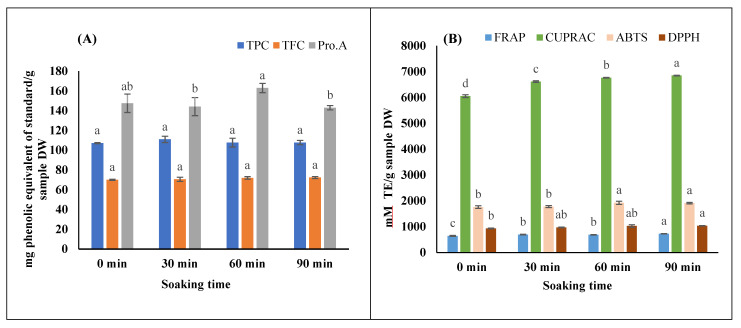
Effect of soaking time on the extraction yield of (**A**) phenolic compounds, and (**B**) antioxidant capacities. The column with same superscript for an individual assay are significantly (*p* < 0.05) different. For TPC, mg phenolic equivalent of standard/g sample DW = mg gallic acid equivalent/g sample in dry weight; for TFC and Pro.A, the mg phenolic equivalent of standard/g sample DW = mg catechin equivalent/g sample in dry weight.

Soaking is known as one of the common conventional extraction techniques [5]. It is also considered a part of other conventional and novel extraction techniques. In this process, dry plant sample tissue structure becomes soft through absorption of solvent; phytochemicals from plant cells and tissue diffuse to solvent. The mass transfer and phytochemical diffusion to or from plant material are accelerated upon applying additional energy/power (i.e., microwave, ultrasound) into the soaked sample. The microwave and ultrasound energy transition to the sample and the extraction is influenced by how well the sample is soaked in MAE and UAE. The time required to soak a sample completely depends on the type of plant tissue and composition, particle size, and structure of plant tissue [29]. In this study, four different soaking times were investigated, and the results are shown in Figure 3.

In general, the effects of soaking time on the extraction of major phenolic groups (TPC, TFC, and Pro.A) was insignificant (*p* > 0.05). However, soaking time significantly influenced the antioxidant properties in the extract. The FRAP and CUPRAC were highest at the soaking time of 90 min. The antioxidant capacities were increased 8–13% for 90 min soaking compared to the control sample. However, soaking time doesnot affected the ABTS and DPPH properties significant. The longer soaking time could help to release additional antioxidants to the extract. However, the longer soaking time did not help extract more phenolics from the LSTT leaves. Therefore, soaking is not needed if phenolics are the target compounds for the extraction of LSTT leaves to save time. The following extraction to test MAE impact, therefore, was conducted without prior soaking of the sample.

### 3.2. Influence of Extraction Parameters on Phenolics and Antioxidants Yield and Fitting of Prediction Models

**Figure 4:**
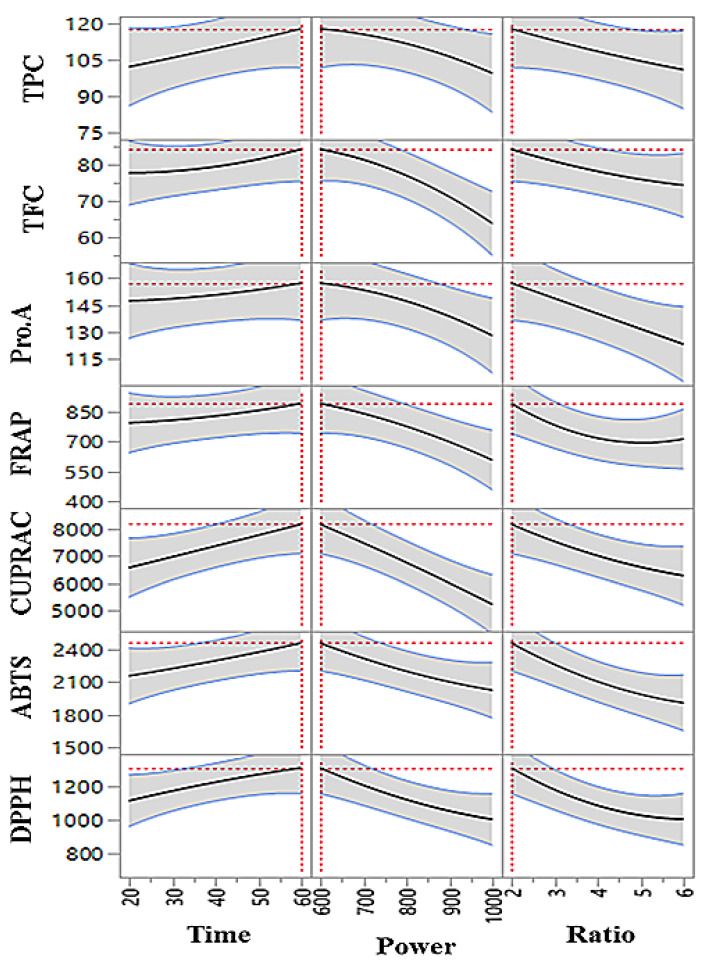
2D contour plots of influence of extraction parameters on phenolic compound and antioxidant capacity.

The individual, interaction, and quadratic terms effects of MAE parameters on the extraction yield of phenolics and antioxidants are presented in Table 2 and Figure 4 2D counterplots. The results show that extraction time positively and significantly (*p* < 0.05) influenced the extraction yield of polyphenols and antioxidant properties.

The extraction time in MAE represents the time length the sample is exposed to microwave irradiation. In a long extraction time, the sample is exposed to microwave irradiation longer which causes more ruptures in sample tissues and plant cell walls leading to an increase in phenolics released [30]. Hence, the extraction yield of the phenolics and antioxidant properties increased in response to increasing the extraction time. The extraction solvent temperature increases with increasing extraction time at a certain microwave power level. The elevated temperature increases the mass diffusion rate and saturation level of the solvent, which also finally results in high phenolics extracted from the sample. However, the extraction rate may decline, or the total extracted phenolic level in the extract may decrease due to thermal degradation [31,32]. The interaction effect of extraction time and microwave power shows a negative influence on the extraction yield of phenolic groups (TPC, TFC, Pro.A) and antioxidant properties (measured by FRAP, CUPRAC, ABTS, and DPPH) (Table 2). The long exposure under high microwave power results in the eventual degradation of phenolics [33]. Liazid et al. [34] also reported significant degradation of various phenolic groups after a certain temperature level rise during MAE. 

From the results, it was found that microwave power and sample to solvent ratio negatively and significantly (*p* < 0.05) influence the extraction yield of all tested phenolic groups and antioxidant capacity measured. This indicates that increasing the value of these two parameters decreases the extraction yield of polyphenols and antioxidant properties in the extract. The microwave generates temperature in polar solvents as a result of dipole rotation [35]. The increasing microwave power level also increases the dipole rotation, which results in temperature elevation of the solvent [36]. The microwave produced localized temperature and pressure, which disseminate to the sample and results in the disruption of the physical structure of sample that speeds up the phytochemical release from plant tissue/cell [37,38]. Hence, the microwave power may help to release more phenolics; however, the high temperature also results in adverse effects on heat-sensitive phenolics and degradation of their properties (i.e., antioxidant) [39,40]. The higher microwave power level and generated high temperature may lead to the breakdown of phenolic compounds or oxidation of the compounds [41]. Like microwave power level, sample to solvent ratio at individual terms also adversely and significantly influences the extraction yield of polyphenols and antioxidant properties in LSTT extract. The increasing sample content in solvent reduces the extraction yields.

Bhuyan et al. [42] also reported similar findings of a negative significant influence of sample to solvent ratio on TPC and TFC in MAE of phenolics. Increasing sample to solvent ratio makes the solvent saturated, resulting in the reduced diffusion rate of phytochemicals from the sample to the solvent. The interaction between microwave power and sample to solvent ratio shows a positive influence on the phenolic groups and antioxidants except for TPC. However, these influences were not significant. 

The interaction and quadratic effects of the MAE parameters show minimal significant effects on the extraction yield of polyphenols and antioxidant properties compared to individual terms. The interaction and quadratic terms effects of the extraction parameters in any specific extraction technique can be different from the individual influence of the parameters. The extraction yield of polyphenols and their bioactivities (i.e., antioxidant) can be similarly or oppositely influenced by interaction or combined effects of extraction parameters compared to the effect of a single factor. The interaction effects of time × power and time × sample to solvent ratio significantly affected the TFC yield, and their influence was negative and positive, respectively. On antioxidant capacity, only CUPRAC was negatively and significantly influenced by effects time × power. Among the quadratic effect, only sample to solvent ratio positively and significantly influence the FRAP and DPPH properties of the extract. 

### 3.3. RSM Model Fitting

RSM analysis produces a polynomial equation, which represents the relationship between the extraction yields and extraction parameters. Table 2 represents ANOVA data, which confirms the reliability of the predictive models. Higher *R*^2^ value, less difference between *R*^2^ and adjusted *R*^2^, lower RMSE value, insignificant lack of fit, and significant *p*-value indicates the models are well fitted with experimental data and can be reliable to predict the values of dependent variables [43,44,45]. In this experiment, for phenolics and antioxidant properties, *R*^2^ values were ranging from 0.92–0.95, lack of fit values was insignificant (*p* > 0.05), and the *p*-value of the model was significant (*p* < 0.05), which indicates that the polynomial prediction models were reliable to predict polyphenols and antioxidant properties level in MAE derived extract. The lower *F* ratio and RMSE values also further confirm their validity. The polynomial equations for TPC, TFC, Pro.A, FRAP, CUPRAC, DPPH, and ABTSare Equations (2)–(8).

(2) $$Y_{\mathrm{TPC}}=41.1724+1.09299X_{1}+0.166X_{2}-9.8967X_{3}-0.00137X_{1}X_{2}+0.05646X_{1}X_{3}-0.003X_{2}X_{3}+0.000745{X_{1}}^{2}-0.000077{X_{2}}^{2}+0.20159{X_{3}}^{2}$$

(3) $$Y_{\mathrm{TFC}}=54.8424+0.2530X_{1}+0.1190178X_{2}-13.493X_{3}-0.00099X_{1}X_{2}+0.1002X_{1}X_{3}+0.0046X_{2}X_{3}+0.0038{X_{1}}^{2}-0.000074{X_{2}}^{2}+0.27893{X_{3}}^{2}$$

(4) $$Y_{\mathrm{Pro.A}}=106.0799+0.57189X_{1}+0.1717X_{2}-13.1057X_{3}-0.001197X_{1}X_{2}0.0297X_{1}X_{3}+0.0047X_{2}X_{3}+0.0047{X_{1}}^{2}-0.00011{X_{2}}^{2}-0.0054{X_{3}}^{2}$$

(5) $$Y_{\mathrm{YFRAP}}=866.8206+5.6347X_{1}+1.1785X_{2}-351.2031X_{3}-0.0145X_{1}X_{2}+1.5888X_{1}X_{3}+0.06806X_{2}X_{3}+0.03667{X_{1}}^{2}-0.0006875{X_{2}}^{2}+21.26395{X_{3}}^{2}$$

(6) $$Y_{\mathrm{CUPRAC}}=7669.0172+109.0059X_{1}+1.14358X_{2}-1647.41889X_{3}-0.128370X_{1}X_{2}+3.21020X_{1}X_{3}+0.945345X_{2}X_{3}+0.017548{X_{1}}^{2}-0.0016675{X_{2}}^{2}+52.2031{X_{3}}^{2}$$

(7) $$Y_{\mathrm{DPPH}}=2125.69072+11.4578X_{1}-1.8545X_{2}-280.392X_{3}-0.0084239X_{1}X_{2}+0.1888X_{1}X_{3}+0.083949X_{2}X_{3}-0.0301{X_{1}}^{2}+0.0008098{X_{2}}^{2}+17.7581{X_{3}}^{2}$$

(8) $$Y_{\mathrm{ABTS}}=3672.6414+11.285346X_{1}-2.47989X_{2}-406.37717X_{3}-0.010661X_{1}X_{2}+0.12491X_{1}X_{3}+0.17535X_{2}X_{3}+0.030003{X_{1}}^{2}+0.00105575{X_{2}}^{2}+19.5607{X_{3}}^{2}$$

### 3.4. Optimization and Validation of Extraction Conditions

RSM analysis offers the best optimized MAE parameters values for the maximum yield of targeted phytochemicals and their desired attributes. Based on the prediction of the models, the optimized MAE conditions were radiation time of 60 min, power of 600 W, and the ratio of 2 g/100 mL. To validate these conditions, the obtained MAE parameters value was applied in a practical experiment and the predicted outputs were compared with experimental yields using a statistical analysis paired comparison *t*-test. The experimental outcomes should be similar to the predicted yield to consider the optimized conditions are valid. The good correlation between the predicted and experimental outputs confirms the reliability of the model and optimized conditions [46]. The phenolic and antioxidant capacities values are presented in Table 3. As shown, there are no significant (*p* < 0.05) differences between the predicted and experimental values for each assay, suggesting that the predicted optimized MAE parameters values were successfully validated and can be used in practical applications. 

### 3.5. Comparison of Extraction Efficiency of Commercial MAE with Other Extraction Methods

The effectiveness of commercial MAE was compared with UAE and conventional SWB in terms of recovery yield of polyphenols and antioxidant properties in dry extract, and the results are shown in Table 4. There is no significant difference in these methods in terms of TPC and TFC levels in the extract. However, Pro.A was significantly higher in MAE-derived extract compared to the extracts from UAE and SWB. Higher in Pro A. can be explained by the more tissue rupture/cell walls under MAE. Hence, more Pro.A is released to the solvent [33,37,47,48,49]. The results (Table 4) also revealed that antioxidant capacity measured by FRAP, ABTS and DPPH were not significantly different between different extraction techniques. However, cupric antioxidant capacity was lower for MAE as compared to UAE or SWB. Overall, there was no significant difference between the three extraction techniques, MAE was two-fold more efficient in solvent consumption than UAE and SWB. Therefore, MAE would be less expensive for solvent and be more economical to remove solvent from the extract when further drying the extracts from lemon-scented tea tree for further applications.

## 4. Conclusions

This study revealed that soaking time before MAE has minimal effect on the extraction yield of polyphenols and antioxidant capacities. However, longer extraction/irradiation time has a positive significant influence on the extraction yields. Other extraction parameters, power, and ratio have considerable but negative effects on the phenolics yield. RSM was successfully applied to optimize the extraction parameters, and the validated optimized extraction conditions are time 60 min, power 600 W, and ratio 2 g/mL. RSM analysis indicates the extraction parameters are more significant compared to interaction and quadratic terms. There was no significant difference in extraction efficiency for phenolics and antioxidant capacity among MAE, UAE, and SWB techniques, but MAE requires less solvent for extraction than UAE and SWB. Therefore, these MAE optimal conditions can be applied for the extraction of polyphenols and antioxidant potentials from lemon-scented tea tree leaves. They can be also employed for recovery polyphenols from spent leaves, which are waste and generated from essential oil production. These extracts enriched with polyphenols can be used as functional ingredients for food and pharmaceutical products.

## Figures and Tables

**Table 1:** Box–Behnken design and observed responses for optimized MAE extraction of polyphenols and antioxidants properties from lemon-scented tea tree.

NR	Extraction Conditions (Independent Variables)				Observed Responses (Dependent Variables) (*n = 3*)						
	Pattern	X_1_	X_2_	X_3_	Phytochemicals			Antioxidant Capacity			
					TPC	TFC	Pro.A	FRAP	CUPRAC	ABTS	DPPH
1	+0+	60	800	6	95.97	72.76	119.24	674.69	5887.81	1822.67	898.86
2	000	40	800	4	93.50	70.72	134.17	618.68	5990.54	1830.26	891.42
3	+−0	60	600	4	110.55	77.81	142.79	723.20	6993.01	2086.81	1075.01
4	−0−	20	800	2	103.55	76.47	145.03	782.86	5958.07	1950.48	976.48
5	−+0	20	1000	4	87.60	63.16	119.58	506.03	5072.00	1689.59	781.74
6	000	40	800	4	96.18	64.00	126.60	489.50	5212.16	1656.59	788.71
7	−0+	20	800	6	80.68	56.34	109.76	427.45	4711.60	1574.48	732.47
8	0+−	40	1000	2	107.85	68.58	135.47	693.84	5677.91	1962.26	989.49
9	0−−	40	600	2	109.67	79.96	146.11	828.69	7540.38	2332.21	1246.04
10	0−+	40	600	6	82.82	59.89	110.10	497.82	5007.44	1721.04	898.86
11	0++	40	1000	6	76.18	55.90	107.12	471.87	4657.52	1631.65	776.63
12	++0	60	1000	4	84.29	60.01	108.38	464.69	4769.93	1818.64	851.45
13	000	40	800	4	99.41	69.14	127.09	588.32	5533.40	1887.12	933.25
14	+0−	60	800	2	109.81	76.86	149.75	775.88	6620.65	2178.68	1112.66
15	−−0	20	600	4	91.89	65.03	134.84	516.74	5241.13	1787.19	870.51

NR (number of runs), − and + represent the lowest and highest value of a parameter respectively. X_1_ (time/extraction time, min), X_2_ (power/microwave power level, W), and X_3_ (ratio/sample to solvent ratio g/100 mL), TPC (mg GAE/g DW), TFC (mg CE/g DW), Pro.A (mg CE/g DW), FRAP (mM TE/g DW), ABTS (mM TE/g DW), DPPH (mM TE/g DW), CUPRAC (mM TE/g DW).

**Table 2:** Regression coefficients of the polynomial model and analysis of variance (ANOVA) results for prediction models fitting.

Model Parameters	Polyphenols			Antioxidant Property Measures			
	TPC	TFC	Pro.A	FRAP	CUPRAC	ABTS	DPPH
**Intercept**							
***β*_0_**	96.37 ***	67.96 ***	129.29 ***	565.50 ***	5578.70 ***	1791.33 ***	871.13 ***
**Linear term**							
***β*_1_**	4.61	3.30 *	1.37	50.67 **	411.07 *	113.13 *	72.09 *
***β*_2_**	−4.88 *	−4.38 **	−7.91 *	−53.75 *	−575.58 **	−103.14 *	−86.39 **
***β*_3_**	−11.90 **	−7.12 **	−16.27 **	−126.18 **	−691.58 **	−209.22 ***	−127.23 **
**Interactions**							
***β*_12_**	−5.49	−3.98 *	−4.79	−61.95	−513.48 *	−42.64	−33.69
***β*_13_**	2.26	4.01 *	1.19	63.55	128.41	4.99	7.55
***β*_23_**	−1.21	1.84	1.91	27.22	378.14	70.14	33.58
**Quadratic**							
***β*_11_**	0.29	1.54	1.68	14.67	7.02	12.01	−12.05
***β*_22_**	−3.07	−2.99	−4.56	−27.50	−66.70	42.23	35.59
***β*_33_**	0.84	1.12	−0.02	85.06 *	208.81	78.24	71.03 *
**Model fitting parameters**							
***R* ^2^**	0.92	0.95	0.92	0.95	0.94	0.94	0.95
**Adjusted *R*^2^**	0.78	0.86	0.78	0.86	0.82	0.84	0.87
**RMSE**	5.34	2.92	6.88	49.92	357.40	84.53	51.03
**Lack of fit**	0.18	0.73	0.22	0.86	0.62	0.91	0.94
***F* ratio of model**	4.77	0.48	3.73	0.24	0.72	0.16	0.12
***P* of model > *F***	0.03	0.001	0.03	0.01	0.02	0.01	0.01

Significantly different at * *p* < 0.05, ** *p* < 0.01, *** *p* < 0.001; *β*_0_: intercept; *β*_1_, *β*_2_, and *β*_3_: linear regression coefficients for time, power, and ratio; *β*_12_, *β*_13_, and *β*_23_: regression coefficients for interaction between time × power, time × ratio, power× ratio; *β*_11_, *β*_22_, and *β*_33_: quadratic regression coefficients for time × time, power × power, and ratio × ratio.

**Table 3:** Justification of the predicted yields of phenolics and antioxidant capacities.

	Values (*n* = 3)	
	Predicted	Experimental
**Phenolic compounds**		
TPC (mg GAE/g DW)	117.85 ± 16.22 ^a^	119.21 ± 7.09 ^a^
TFC (mg CE/g DW)	84.25 ± 8.86 ^a^	85.31 ± 4.55 ^a^
Pro.A (mg CE/g DW)	157.41 ± 20.95 ^a^	137.51 ± 12.52 ^a^
**Antioxidant capacities**		
FRAP (mM TE/g DW)	893.95 ± 151.6 ^a^	834.62 ± 187.68 ^a^
CUPRAC (mM TE/g DW)	8169.28 ± 1085.42 ^a^	7369.63 ± 834.32 ^a^
DPPH (mM TE/g DW)	1311.16 ± 155 ^a^	1306.93 ± 129.77 ^a^
ABTS (mM TE/g DW)	2457.09 ± 256.72 ^a^	2297.36 ± 220.98 ^a^

All the values are means ± standard deviations and those in the same row sharing the same superscript letter are not significantly different from each other (*p* < 0.05).

**Table 4:** Phytochemical and antioxidant content in dried extract from three different extraction methods.

	MAE	UAE	SWB
**Phenolic compounds**			
TPC (mg GAE/g DW)	323.99 ± 4.35 ^a^	317.45 ± 9.30 ^a^	308.46 ± 7.12 ^a^
TFC (mg CE/g DW)	220.14 ± 3.43 ^a^	216.47 ± 3.89 ^a^	212.37 ± 4.26 ^a^
Pro.A (mg CE/g DW)	325.22 ± 5.45 ^a^	301.54 ± 4.54 ^b^	293.02 ± 1.85 ^b^
**Antioxidant capacities**			
FRAP (mM TE/g DW)	3258.86 ± 81.47 ^a^	3339.85 ± 164.35 ^a^	3321.52 ± 102.98 ^a^
CUPRAC (mM TE/g DW)	23,069.50 ± 90.20 ^c^	24,123.43 ± 151.70 ^b^	24,887.95 ± 156.65 ^a^
ABTS (mM TE/g DW)	7860.74 ± 38.64 ^a^	7790.72 ± 58.07 ^a^	7761.92 ± 371.43 ^a^
DPPH (mM TE/g DW)	2541.59 ± 27.45 ^a^	2544.28 ± 72.13 ^a^	2465.75 ± 63.69 ^a^

All the values are means ± standard deviations and those in the same row sharing the same superscript letter are not significantly different from each other (*p* < 0.05).

## Data Availability

Data is contained within this article.

## References

[1] Harjo B., Wibowo C., Ng K.M. (2004). Development of Natural Product Manufacturing Processes: Phytochemicals. Chem. Eng. Res. Des..

[2] Albuquerque B.R., Heleno S.A., Oliveira M.B.P.P., Barros L., Ferreira I.C.F.R. (2020). Phenolic compounds: Current industrial applications, limitations and future challenges. Food Funct..

[3] Martillanes S., Rocha-Pimienta J., Cabrera-Bañegil M., Martín-Vertedor D., Delgado-Adámez J. (2017). Application of phenolic compounds for food preservation: Food additive and active packaging. Phenolic Compounds–Biological Activity.

[4] Saifullah M., McCullum R., Vuong Q.V. (2021). Development of Ultrasound-assisted Extraction Conditions for the Optimal Yield of Phenolic Compounds and Antioxidant Properties from Lemon Myrtle (*Backhousia citriodora*) Leaves. Curr. Nutraceuticals.

[5] Alara O.R., Abdurahman N.H., Ukaegbu C.I. (2018). Soxhlet extraction of phenolic compounds from Vernonia cinerea leaves and its antioxidant activity. J. Appl. Res. Med. Aromat. Plants.

[6] Tatke P., Rajan M. (2014). Comparison of Conventional and Novel Extraction Techniques for the Extraction of Scopoletin from Convolvulus Pluricaulis. Indian J. Pharm. Educ. Res..

[7] Zhao C.-N., Zhang J.-J., Li Y., Meng X., Li H.-B. (2018). Microwave-Assisted Extraction of Phenolic Compounds from Melastoma sanguineum Fruit: Optimization and Identification. Molecules.

[8] Sanchez-Reinoso Z., Mora-Adames W.I., Fuenmayor C.A., Darghan-Contreras A.E., Gardana C., Gutiérrez L.-F. (2020). Microwave-assisted extraction of phenolic compounds from Sacha Inchi shell: Optimization, physicochemical properties and evaluation of their antioxidant activity. Chem. Eng. Processing Process Intensif..

[9] Alara O.R., Abdurahman N.H., Ukaegbu C.I. (2021). Extraction of phenolic compounds: A review. Curr. Res. Food Sci..

[10] Yuan Y., Macquarrie D.J. (2015). Microwave assisted step-by-step process for the production of fucoidan, alginate sodium, sugars and biochar from Ascophyllum nodosum through a biorefinery concept. Bioresour. Technol..

[11] Quiles-Carrillo L., Mellinas C., Garrigos M.C., Balart R., Torres-Giner S. (2019). Optimization of Microwave-Assisted Extraction of Phenolic Compounds with Antioxidant Activity from Carob Pods. Food Anal. Methods.

[12] Esquivel-Hernández D.A., Ibarra-Garza I.P., Rodríguez-Rodríguez J., Cuéllar-Bermúdez S.P., Rostro-Alanis M.D.J., Alemán-Nava G.S., García-Pérez J.S., Parra-Saldívar R. (2016). Green extraction technologies for high-value metabolites from algae: A review. Biofuels Bioprod. Biorefining.

[13] Kala H.K., Mehta R., Sen K.K., Tandey R., Mandal V. (2016). Critical analysis of research trends and issues in microwave assisted extraction of phenolics: Have we really done enough. TrAC Trends Anal. Chem..

[14] Chan C.-H., Yusoff R., Ngoh G.-C., Kung F.W.-L. (2011). Microwave-assisted extractions of active ingredients from plants. J. Chromatogr. A.

[15] Nuerxiati R., Abuduwaili A., Mutailifu P., Wubulikasimu A., Rustamova N., Jingxue C., Aisa H.A., Yili A. (2019). Optimization of ultrasonic-assisted extraction, characterization and biological activities of polysaccharides from Orchis chusua D. Don (Salep). Int. J. Biol. Macromol..

[16] Das A.K., Dewanjee S., Sarker S.D., Nahar L. (2018). Chapter 3—Optimization of Extraction Using Mathematical Models and Computation. Computational Phytochemistry.

[17] Saifullah M., McCullum R., McCluskey A., Van Vuong Q. (2021). Effect of drying techniques and operating conditions on the retention of color, phenolics, and antioxidant properties in dried lemon scented tea tree (*Leptospermum petersonii*) leaves. J. Food Process. Preserv..

[18] Afolabi W.O., Hussein A., Shode F.O., Le Roes-Hill M., Rautenbach F. (2020). *Leptospermum petersonii* as a Potential Natural Food Preservative. Molecules.

[19] Saifullah M., McCullum R., McCluskey A., Vuong Q. (2020). Comparison of conventional extraction technique with ultrasound assisted extraction on recovery of phenolic compounds from lemon scented tea tree (*Leptospermum petersonii*) leaves. Heliyon.

[20] Bezerra M.A., Santelli R.E., Oliveira E.P., Villar L.S., Escaleira L.A. (2008). Response surface methodology (RSM) as a tool for optimization in analytical chemistry. Talanta.

[21] Candioti L.V., De Zan M.M., Cámara M.S., Goicoechea H.C. (2014). Experimental design and multiple response optimization. Using the desirability function in analytical methods development. Talanta.

[22] AOCS (1990). Official Methods and Recommended Practices of the American Oil Chemicsts’ Society.

[23] Zhuang X.P., Lu Y.Y., Yang G.S. (1992). Extraction and determination of flavonoid in Ginkgo. Chin. Herb. Med..

[24] Sun B.S., Ricardo-Da-Silva J.M., Spranger M.I. (1998). Critical factors of vanillin assay for catechins and proanthocyanidins. J. Agric. Food Chem..

[25] Benzie I.F., Strain J.J. (1996). The ferric reducing ability of plasma (FRAP) as a measure of “antioxidant power”: The FRAP assay. Anal. Biochem..

[26] Brand-Williams W., Cuvelier M.E., Berset C. (1995). Use of a free radical method to evaluate antioxidant activity. LWT-Food Sci. Technol..

[27] Arnao M.B., Cano A., Acosta M. (2001). The hydrophilic and lipophilic contribution to total antioxidant activity. Food Chem..

[28] Apak R., Güçlü K., Özyürek M., Karademir S.E. (2004). Novel Total Antioxidant Capacity Index for Dietary Polyphenols and Vitamins C and E, Using Their Cupric Ion Reducing Capability in the Presence of Neocuproine:  CUPRAC Method. J. Agric. Food Chem..

[29] Witrowa-Rajchert D., Lewicki P.P. (2006). Rehydration properties of dried plant tissues. Int. J. Food Sci. Technol..

[30] Xiaokang W., Lyng J., Brunton N.P., Cody L., Jacquier J.-C., Harrison S.M., Papoutsis K. (2020). Monitoring the effect of different microwave extraction parameters on the recovery of polyphenols from shiitake mushrooms: Comparison with hot-water and organic-solvent extractions. Biotechnol. Rep..

[31] Brglez Mojzer E., Knez Hrnčič M., Škerget M., Knez Ž., Bren U. (2016). Polyphenols: Extraction Methods, Antioxidative Action, Bioavailability and Anticarcinogenic Effects. Molecules.

[32] Shao P., He J., Sun P., Zhao P. (2011). Analysis of conditions for microwave-assisted extraction of total water-soluble flavonoids from Perilla Frutescens leaves. J. Food Sci. Technol..

[33] Kumar M., Dahuja A., Tiwari S., Punia S., Tak Y., Amarowicz R., Bhoite A.G., Singh S., Joshi S., Panesar P.S. (2021). Recent trends in extraction of plant bioactives using green technologies: A review. Food Chem..

[34] Liazid A., Palma M., Brigui J., Barroso C.G. (2007). Investigation on phenolic compounds stability during microwave-assisted extraction. J. Chromatogr. A.

[35] Bart J.C. (2005). Chapter-3 Sample preparation perspectives. Additives in Polymers: Industrial Analysis and Applications.

[36] Alara O.R., Abdurahman N.H. (2019). Microwave-assisted extraction of phenolics from Hibiscus sabdariffa calyces: Kinetic modelling and process intensification. Ind. Crop. Prod..

[37] Vu H.T., Scarlett C.J., Vuong Q. (2019). Maximising recovery of phenolic compounds and antioxidant properties from banana peel using microwave assisted extraction and water. J. Food Sci. Technol..

[38] Hayat K., Hussain S., Abbas S., Farooq U., Ding B., Xia S., Jia C., Zhang X., Xia W. (2009). Optimized microwave-assisted extraction of phenolic acids from citrus mandarin peels and evaluation of antioxidant activity in vitro. Sep. Purif. Technol..

[39] Saifullah M., McCullum R., Vuong Q. (2020). Maximising extraction yields of gallic acid and hesperetin from lemon myrtle (*Backhousia citriodora*) leaf using microwave assisted extraction. Results Chem..

[40] Dahmoune F., Nayak B., Moussi K., Remini H., Madani K. (2015). Optimization of microwave-assisted extraction of polyphenols from *Myrtus communis* L. leaves. Food Chem..

[41] Le X.D., Nguyen M.C., Vu D.H., Pham M.Q., Pham Q.L., Nguyen Q.T., Nguyen T.A., Pham V.T., Bach L.G., Van Nguyen T. (2019). Optimization of Microwave-Assisted Extraction of Total Phenolic and Total Flavonoid Contents from Fruits of Docynia indica (Wall.) Decne. Using Response Surface Methodology. Processes.

[42] Bhuyan D.J., Van Vuong Q., Chalmers A.C., van Altena I.A., Bowyer M.C., Scarlett C.J. (2015). Microwave-assisted extraction of Eucalyptus robusta leaf for the optimal yield of total phenolic compounds. Ind. Crop. Prod..

[43] Rodsamran P., Sothornvit R. (2019). Extraction of phenolic compounds from lime peel waste using ultrasonic-assisted and microwave-assisted extractions. Food Biosci..

[44] Belwal T., Bhatt I.D., Rawal R.S., Pande V. (2016). Microwave-assisted extraction (MAE) conditions using polynomial design for improving antioxidant phytochemicals in *Berberis asiatica* Roxb. ex DC. leaves. Ind. Crop. Prod..

[45] Gammoudi N., Mabrouk M., Bouhemda T., Nagaz K., Ferchichi A. (2021). Modeling and optimization of capsaicin extraction from Capsicum annuum L. using response surface methodology (RSM), artificial neural network (ANN), and Simulink simulation. Ind. Crop. Prod..

[46] Ali A., Lim X.Y., Chong C.H., Mah S.H., Chua B.L. (2018). Optimization of ultrasound-assisted extraction of natural antioxidants from Piper betle using response surface methodology. LWT.

[47] Kumar M., Dahuja A., Sachdev A., Kaur C., Varghese E., Saha S., Sairam K. (2019). Evaluation of enzyme and microwave-assisted conditions on extraction of anthocyanins and total phenolics from black soybean (*Glycine max* L.) seed coat. Int. J. Biol. Macromol..

[48] Pinela J., Prieto M., Carvalho A.M., Barreiro M.F., Oliveira M.B.P., Barros L., Ferreira I.C. (2016). Microwave-assisted extraction of phenolic acids and flavonoids and production of antioxidant ingredients from tomato: A nutraceutical-oriented optimization study. Sep. Purif. Technol..

[49] Gouda M., El-Din Bekhit A., Tang Y., Huang Y., Huang L., He Y., Li X. (2021). Recent innovations of ultrasound green technology in herbal phytochemistry: A review. Ultrason. Sonochem..

